# Graph theoretical analysis of evoked potentials shows network influence of epileptogenic mesial temporal region

**DOI:** 10.1002/hbm.25418

**Published:** 2021-06-24

**Authors:** Mark A. Hays, Christopher Coogan, Nathan E. Crone, Joon Y. Kang

**Affiliations:** ^1^ Department of Biomedical Engineering Johns Hopkins University School of Medicine Baltimore Maryland USA; ^2^ Department of Neurology Johns Hopkins University School of Medicine Baltimore Maryland USA

**Keywords:** evoked potential, graph theory, intracranial EEG, mesial temporal lobe epilepsy, single‐pulse electrical stimulation

## Abstract

It is now widely accepted that seizures arise from the coordinated activity of epileptic networks, and as a result, traditional methods of analyzing seizures have been augmented by techniques like single‐pulse electrical stimulation (SPES) that estimate effective connectivity in brain networks. We used SPES and graph analytics in 18 patients undergoing intracranial EEG monitoring to investigate effective connectivity between recording sites within and outside mesial temporal structures. We compared evoked potential amplitude, network density, and centrality measures inside and outside the mesial temporal region (MTR) across three patient groups: focal epileptogenic MTR, multifocal epileptogenic MTR, and non‐epileptogenic MTR. Effective connectivity within the MTR had significantly greater magnitude (evoked potential amplitude) and network density, regardless of epileptogenicity. However, effective connectivity between MTR and surrounding non‐epileptogenic regions was of greater magnitude and density in patients with focal epileptogenic MTR compared to patients with multifocal epileptogenic MTR and those with non‐epileptogenic MTR. Moreover, electrodes within focal epileptogenic MTR had significantly greater outward network centrality compared to electrodes outside non‐epileptogenic regions and to multifocal and non‐epileptogenic MTR. Our results indicate that the MTR is a robustly connected subnetwork that can exert an overall elevated propagative influence over other brain regions when it is epileptogenic. Understanding the underlying effective connectivity and roles of epileptogenic regions within the larger network may provide insights that eventually lead to improved surgical outcomes.

## Graphical abstract

Hays et al. utilize evoked potentials from single‐pulse electrical stimulation to map effective networks in mesial temporal epilepsy patients. Graph theoretical analysis of these connections is applied to characterize the role of mesial temporal structures within broader epileptogenic networks, revealing a highly hyperexcitable and influential densely connected subnetwork. This approach provides additional groundwork to better understand epilepsy as a network disorder and identify epileptogenic hubs of connectivity for network targeted treatments.

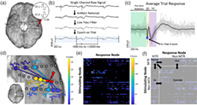

**Abbreviations**
ECoG: electrocorticography
mTLE: mesial temporal lobe epilepsy
MTR: mesial temporal region
S‐EEG: stereoelectroencephalography
SOZ: seizure onset zone
SPES: single‐pulse electrical stimulation

## 1 INTRODUCTION

Epilepsy is the world's most common chronic brain disorder, affecting some 70 million people worldwide (Singh & Trevick, 2016). Roughly, a third of these people have seizures resistant to drug therapy and are potential candidates for a growing number of advanced surgical therapies (Kalilani, Sun, Pelgrims, Noack‐Rink, & Villanueva, 2018). However, there is a growing appreciation that to be effective, these therapies must be guided not only by localization of the ictal onset zone, but also by a more comprehensive map of its effective connectivity with sites in a broader epileptogenic network responsible not only for seizure propagation, but also for ictogenesis.

Single‐pulse electrical stimulation (SPES) has been increasingly used to estimate connectivity between sites recorded with intracranial electrodes for the surgical management of intractable focal epilepsy. SPES elicits electrophysiological responses in regions that are effectively connected to the stimulation site. Compared to other connectivity measures such as resting functional magnetic resonance imaging and diffusion tensor imaging, SPES provides additional directional information about the corticocortical or subcortical interactions. Prior studies demonstrate that SPES can delineate epileptogenic networks by evoking spontaneous delayed responses (Valentín et al., 2005) or eliciting large amplitude evoked potentials (Enatsu, Jin, et al., 2012; Enatsu, Piao, et al., 2012; Iwasaki et al., 2010; Parker et al., 2018) and time‐frequency single pulse‐evoked fast ripples (van 't Klooster et al., 2011). A few investigators have applied graph analytics to quantitatively interpret the directed connections of the seizure networks (Keller et al., 2014; Parker et al., 2018; van Blooijs, Leijten, van Rijen, Meijer, & Huiskamp, 2018; Zhao et al., 2019) with highly variable findings, most likely due to the heterogeneous spatial sampling and limited stimulation in a small number of subjects.

In the present study, we utilize SPES to explore alterations of network effective connectivity of eighteen patients with intractable mesial temporal lobe epilepsy (mTLE), the most common type of surgically remediable epilepsy (Engel Jr, 2001). By employing a graph theoretical‐based approach to analyze the causal interactions mapped by SPES, we aimed to understand the role of mesial temporal structures within a broader epileptogenic network, which we assumed could change with varying degrees of epileptogenicity across patients and across hemispheres within the same patient. We propose that identifying how epileptogenic and non‐epileptogenic mesial temporal structures differ in their influence within the effective network can help characterize how electrophysiological interactions are altered between epileptogenic regions and rest of the brain. This network‐based understanding can offer insight into mechanisms underlying seizure generation and propagation, and potentially provide a mechanistic framework for a targeted treatment approach in determining the importance of regions within the context of the epileptogenic network.

## 2 METHODS

### 2.1 Patients

We studied eighteen patients who were undergoing intracranial EEG monitoring prior to surgery for treatment of drug‐resistant epilepsy at the Johns Hopkins Epilepsy Center from January 2016 through March 2020. All patients had EEG implantation and stimulation that included at least one mesial temporal lobe. None of the patients had visible lesions seen on the mesial temporal structures such as a tumor, dysplasia, or mesial temporal sclerosis. The study was approved by the Johns Hopkins School of Medicine Institutional Review Board (IRB 00247294) and was conducted using guidelines established in accordance with the Code of Ethics of the World Medical Association (1964, Declaration of Helsinki).

### 2.2 Electrode placement

Patients were implanted with surface electrocorticography (ECoG) electrode grids and/or stereoelectroencephalography (S‐EEG) electrodes. The type, number, and location of the electrodes were determined by the suspected location of the epileptogenic zone in each patient according to noninvasive tests including clinical seizure history, neuroimaging, neuropsychology, and scalp EEG recordings. Patients underwent the S‐EEG procedure if: (a) the suspected seizure onset zone (SOZ) was in deep‐seated locations such as the mesial structures of the temporal lobe but imaging (MRI or PET) was non‐lesional, (b) failure of previous subdural invasive studies to clearly outline the exact location of SOZ, (c) there was a need for bilateral exploration for possible bilateral independent seizure onset, or (d) there was concern for dual pathology or multifocal epilepsy.

ECoG electrodes consisted of arrays of macroelectrodes [2.3 mm exposed diameter, 1 cm spacing, AdTech (Racine, WI) or PMT Corp. (Chanhassen, MN)]. S‐EEG depth electrodes (AdTech, Racine, WI) were implanted stereotactically using ROSA robotic assistant device (Medtech, Montpellier, France) as part of standard patient care. These depths were multi‐contact and consisted of 6–10 cylindrical 2.3 mm long platinum contacts separated by 5 mm between centers of adjacent electrodes of the same bundle.

### 2.3 Electrode localization

Final electrode locations were obtained by combining information from post‐implantation CT and brain MRI using BioImage Suite (Duncan et al., 2004). Using FreeSurfer (Fischl, 2012) parcellation and visual verification with post‐implant MRI, electrodes within amygdala, hippocampus, entorhinal cortex, or parahippocampal gyrus were identified as our region of interest within the mesial temporal lobe and will be referred to here as the mesial temporal region (MTR).

### 2.4 EEG recording and determination of seizure onset

ECoG and S‐EEG recordings for clinical review were performed with a Neurofax EEG‐1100 amplifier (Nihon Kohden, Tokyo, Japan), digitized at 2 kHz with 16‐bit resolution, and 0.016–300 Hz band‐pass filtered. All patients in the current sample had at least one typical seizure captured during their stay in the Epilepsy Monitoring Unit. Ictal EEG data was reviewed by at least two board‐certified epileptologists (NEC, JYK) to identify the SOZ. Patients were divided into three groups based on the involvement of the amygdala, hippocampus, entorhinal cortex, and parahippocampal gyrus in the SOZ: (a) focal epileptogenic MTR: SOZ is isolated to unilateral MTR, (b) multifocal epileptogenic MTR: unilateral MTR is involved in seizure onset but SOZ extends beyond unilateral MTR, (c) non‐epileptogenic MTR: MTR is not involved in seizure onset but is ipsilateral to the SOZ.

### 2.5 SPES data acquisition and pre‐processing

**FIGURE 1:**
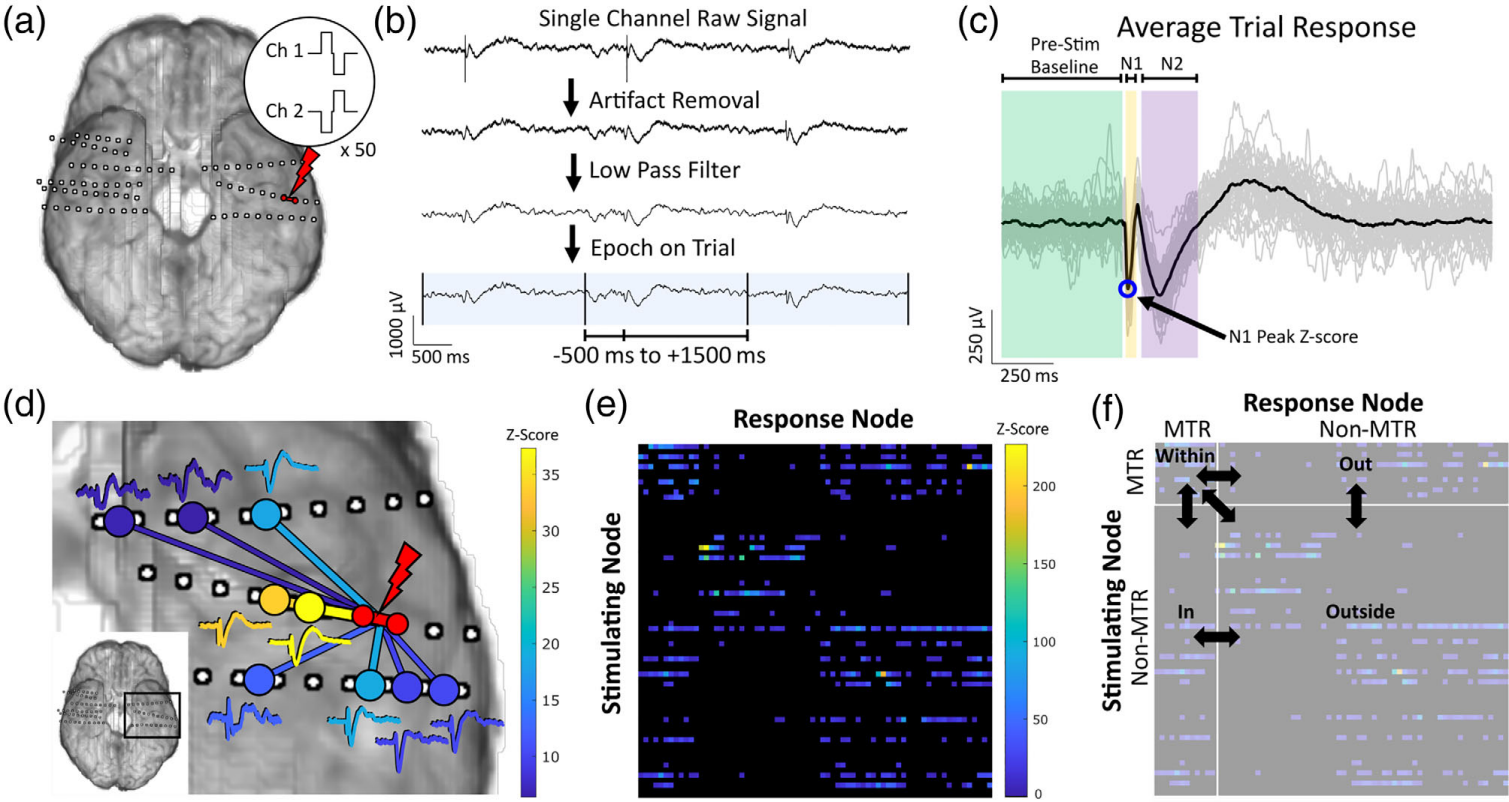
Experimental methods. (a) Single‐pulse electrical stimulation (SPES) using a biphasic 0.3 ms pulse is applied in a bipolar manner to adjacent electrodes for 50 trials at an interstimulus interval (ISI) of 1 or 2 s. Example pulse waveforms applied to the stimulating electrodes are pictured. (b) Raw signal recordings of each channel are pre‐processed before response analysis. After re‐referencing using a bipolar montage, stimulation artifacts within −5 to 10 ms relative to stimulus are removed, and a 50 Hz low pass filter is applied. Trials are selected using analysis windows of −500 to 1,500 ms for 2 s ISI (−250 to 750 ms for 1 s ISI). (c) Trials are centered using baseline (−500 to −5 ms for 2 s ISI, −250 to −5 ms for 1 s ISI) mean and averaged. The average response is normalized by the baseline standard deviation, and amplitude of the N1 peak within 10 to 50 ms is used to quantify the signal's *Z*‐score. (d) For each stimulated pair of electrodes, average responses with a *Z*‐score greater than 6 are considered significant evoked potentials, representing a causal electrophysiological relationship between the stimulation and response sites. Significant responses to one stimulation are shown here as an example, colored according to the magnitude of the *Z*‐score. (e) With multiple pairs of electrodes stimulated, the *Z*‐score responses of all channels from each stimulation block becomes one row in the adjacency matrix of the weighted, directed graph of effective connectivity. The magnitude of the causal relationship between a stimulating site and response site is quantified by the *Z*‐score at the row of the stimulating site and the column of the response site, shown here by color intensity. (f) Subnetworks are grouped based on location of stimulating and response sites with respect to mesial temporal region (MTR), either *within* (stimulate MTR, response in MTR), *in* (stimulate outside MTR, response in MTR), *out* (stimulate MTR, response outside MTR), or *outside* (stimulate outside MTR, response outside MTR). The properties of each subnetwork are calculated and compared to that of the others. MTR, mesial temporal region

ECoG and S‐EEG signals for SPES analysis were recorded using a NeuroPort amplifier (Blackrock Microsystems, Salt Lake City, UT), filtered (analog Butterworth antialiasing filters: first‐order high‐pass at 0.3 Hz, third‐order low‐pass at 7500 Hz), digitized at 16‐bit resolution, and down‐sampled to 1 kHz with a digital antialiasing filter. SPES with a CereStim R96 (Blackrock Microsystems, Salt Lake City, UT) was applied in a bipolar manner to adjacent electrodes. At each stimulation site, 50 biphasic pulses with 0.3 ms pulse width were applied (Figure 1a) with an interstimulus interval (ISI) of 1 or 2 s. Both intervals provided sufficient time for response channels to return to baseline. Stimulation current intensity was first set using a titration procedure, titrating 0.5–1 mA increments while watching for after‐discharges, until evoked potentials (EP) were seen consistently during real‐time visualization, up to a maximum of 10 mA with charge density well within safety limits (<50 μC/cm^2^) (Gordon et al., 1990). The mean and variance of the final current intensities of stimulations, separated by location with respect to MTR and SOZ in each patient, are detailed in Table S1.

Following rejection of channels with excessive noise, electrode channels were re‐referenced using a bipolar montage. An artifact removal procedure that preserves the time‐frequency composition of the surrounding signal (Crowther et al., 2019; Huang et al., 2019) replaced artifactual data from −5 to 10 ms relative to stimulus with reversed, tapered copies of the signals surrounding this period. Following artifact removal, the signals were low‐pass filtered (50 Hz). All preprocessing and analysis of SPES results were performed using custom scripts in Matlab (R2019b, MathWorks, Natick, MA).

### 2.6 Evoked potential calculations

For each pair of electrodes stimulated, an analysis window time‐locked to the stimulus (−500 ms to 1,500 ms for 2 ISI, −250 ms to 750 ms for 1 s ISI) was used to compute the average responses (Figure 1b). The mean of the pre‐stimulus baseline (−500 ms to −10 ms for 2 s ISI, −250 to −10 ms for 1 s ISI) was subtracted to baseline‐center each response before averaging across all responses for each channel. The average response was divided by the standard deviation of the pre‐stimulus baseline, to obtain a normalized average response. The typical morphology of EPs comprises early (N1) and late (N2) negative deflections, typically occurring between 10 to 50 ms and 70 to 300 ms post‐stimulus respectively (Matsumoto et al., 2004). Peak detection was used to identify the latency, magnitude, and polarity of N1 and N2 potentials in the normalized average response for each channel (Figure 1c). Since the N1 potential is considered to represent direct excitatory connectivity, we used the absolute value of the N1 peak amplitude to quantify the magnitude of the evoked response and defined this as the channel's *Z*‐score. Responses with a *Z*‐score greater than 6 were considered significant (Keller et al., 2011), and we used these normalized response amplitudes to quantify the effective connectivity between the stimulation and response sites (Figure 1d). The *Z*‐scores between all stimulation and response sites were used as edge weights between the electrodes as nodes in weighted, directed networks for graph theoretical analysis (Figure 1e).

### 2.7 Evoked potential distribution, strength, and density

To test how epileptogenicity of the MTR affects the distributions of its effective connections, we compared the ratio of responses inside and outside the MTR and the proportion of possible responses in each region when stimulating from within the MTR ipsilateral versus contralateral to seizure onset. This was done for each bilaterally stimulated patient, and statistical significance was calculated using Fisher's exact test. Pooled responses over each patient group were also used to compare the MTR ipsilateral versus contralateral to seizure onset at the group level, using chi‐squared tests.

Stimulation‐response pairs were classified into four different categories, based on the location of stimulation and response sites: *within*, stimulation and response both inside MTR; *out*, stimulation inside MTR and response outside MTR; *in*, stimulation outside MTR and response inside MTR; *outside*, stimulation and response both outside MTR (Figure 1f). Connections involving the SOZ outside of MTR were not included, so that the only variance in epileptogenicity occurs within MTR. For bilaterally stimulated patients, connections were also grouped into these categories with respect to the contralateral non‐epileptogenic MTR. We analyzed the *Z*‐scores and weighted density of the subnetworks formed by each connection type described above. Weighted density is calculated as the sum of the connection weights (*Z*‐scores) divided by the number of possible connections given the number and location of stimulations and can be considered a measure of the average *Z*‐score of each subnetwork.

### 2.8 Centrality measures

Graph theoretical measures of centrality can quantify the importance of nodes within the context of a larger network, and each type of centrality can inform a different aspect of each node's network properties. Degree centrality has been commonly used to characterize graphs derived from EPs (Keller et al., 2014; Parker et al., 2018; van Blooijs et al., 2018; Zhao et al., 2019). The two variants, indegree and outdegree, are calculated as the sum of incoming or outgoing edge weights, respectively, for each node in the network. Since the edge weights in effective networks are the EP magnitudes, these measures can capture both the distribution and strength of the direct electrophysiological connections of each node. Hyperlink‐induced topic search (HITS) centrality (Kleinberg, 1999) gives nodes authority and hub scores, where important authorities receive projections from important hubs, and important hubs project to important authorities. While originally designed to find a small number of authoritative sources and hubs of information, these measures may be applied to effective networks to identify a small number of nodes that are particularly responsible for projecting or receiving the largest EP. Katz centrality (Katz, 1953) factors in edge weights between nodes more than one edge away and gives nodes more importance for having connections with other important nodes. This can provide a unique characterization of the effective network because it allows indirect connections to influence a node's centrality. The two forms, Katz‐receive and Katz‐broadcast, rely on incoming and outgoing connections, respectively, to quantify a node's ability to receive or broadcast information.

Each centrality measure was calculated for every electrode node in each patient's network and normalized prior to comparison across patients. Degree centralities were normalized by the number of possible connections each node could have received or projected, and HITS and Katz centralities were each normalized to unit vectors. For comparisons, electrodes for each patient were grouped by location: within the MTR ipsilateral to seizure onset, within the non‐epileptogenic MTR contralateral to the seizure onset, or in non‐epileptogenic regions outside of both MTRs. Since nodes that were not stimulated will have insignificant outgoing centrality, only stimulated electrode centrality measures were included.

### 2.9 Statistical analysis

To investigate how epileptogenicity affects the effective connectivity of the MTR with the rest of the brain, we compared *Z*‐scores and weighted density across the factors patient group and connection type (and laterality for bilateral patients), and centrality across the factors patient group and electrode location. The *Z*‐scores of every significant response and the centrality measures of every stimulated node were each pooled within patient group and compared using non‐parametric tests due to the non‐normality of the data. This analysis also allows for analogous comparisons to similar studies that used non‐parametric methods on pooled patient evoked response amplitudes and node centrality (Parker et al., 2018; van Blooijs et al., 2018). Grouped Kruskal‐Wallis tests were used to determine the presence of simple effects for each factor, followed by *post hoc* Dunn's tests for pairwise differences in medians when significant simple effects were observed.

A similar procedure was used for analyzing density and averaged centrality values. For each metric, the data was fit with a linear mixed effects model with fixed effects of patient group and electrode location for average centrality, and fixed effects of patient group and connection type (and laterality) for the densities, both with patient number as a random effect. The residuals of each model were checked for reasonable normality using Shapiro–Wilk tests. Interaction and main effects were tested using likelihood ratio tests (LRT) comparing full models to reduced models. Because of our a priori interest in how each patient group and location affects each connectivity metric, tests for simple effects using LRTs were conducted regardless of a statistically significant interaction. If a significant simple effect was observed, *post hoc* pairwise comparisons were performed to investigate differences between groups. These were calculated with two‐tailed *t*‐tests using the pooled estimate of standard error from the model and containment degrees of freedom.

All reported *p*‐values were false‐discovery rate (FDR) corrected for multiple comparisons accordingly, with adjusted *p* < .05 considered significant (Benjamini & Hochberg, 1995). Statistical calculations were computed in R (R Core Team, 2019) with additional packages nlme (Pinheiro et al., 2020) and emmeans (Lenth et al., 2020).

## 3 RESULTS

Eighteen patients (eight males, ten females, median age 33.5, range: 19–62) were included in this study (Table 1). All patients underwent S‐EEG implantation of at least one structure within the MTR. Four patients had focal seizure onset isolated to the MTR, nine patients had multifocal seizure onset with involvement of the MTR, and five patients did not have seizure onset in the MTR. Eight patients underwent unilateral mesial temporal stimulation, and ten patients underwent bilateral mesial temporal stimulation. A median of 100.5 electrodes were implanted (range: 66–214) and 35.5 electrodes were stimulated (range: 20–53) per patient. Within the MTR, a median of 9 electrodes were implanted (range: 5–26), and a median of 6 electrodes were stimulated (range: 2–12).

**TABLE 1:** Patient characteristics

Patient no.	Gender	Age	Implant type	No. of electrodes implanted (Stimulated)	Implanted mesial temporal structures		No. of electrodes implanted (Stimulated) in mesial temporal structures		Patient group
					Left	Right	Left	Right	
P1	M	62	S‐EEG	106 (28)	A^a^,H^a^	A^a^,H^a^	7 (4)	7 (5)	Multifocal epileptogenic MTR
P2	M	26	Grid, strip, S‐EEG	136 (24)	A^a^,H^a^,E^a^	A^a^,H^a^,E^a^,P	14 (6)	26 (6)	Multifocal epileptogenic MTR
P3	F	26	S‐EEG	134 (44)	A^a^,E,P^a^	A^a^,P^a^	9 (4)	9 (8)	Multifocal epileptogenic MTR
P4	F	24	S‐EEG	140 (37)	A^a^,H^a^	None	13 (10)	0 (0)	Non‐epileptogenic MTR
P5	F	39	S‐EEG	92 (47)	A^a^,H^a^	H^a^	7 (5)	9 (6)	Multifocal epileptogenic MTR
P6	F	26	S‐EEG	98 (38)	A^a^,H	None	13 (4)	0 (0)	Non‐epileptogenic MTR
P7	F	42	Grid, S‐EEG	214 (18)	A^a^,H^a^,E,P	A,H,E,P	22 (6)	0 (0)	Multifocal epileptogenic MTR
P8	M	48	S‐EEG	103 (34)	H^a^	H^a^	5 (4)	7 (4)	Multifocal epileptogenic MTR
P9	F	23	S‐EEG	108 (35)	A^a^,H^a^	A^a^,H^a^	11 (10)	7 (4)	Focal epileptogenic MTR
P10	F	23	S‐EEG	176 (53)	A^a^,H^a^,E	A^a^,H^a^,E,P^a^	10 (4)	14 (6)	Non‐epileptogenic MTR
P11	M	51	S‐EEG	114 (20)	A,H,P	A^a^,H^a^,P	10 (0)	11 (10)	Multifocal epileptogenic MTR
P12	M	32	S‐EEG	92 (26)	A	A,H^a^	5 (0)	9 (4)	Multifocal epileptogenic MTR
P13	M	19	S‐EEG	84 (36)	A^a^,H^a^,E^a^,P^a^	None	19 (8)	0 (0)	Focal epileptogenic MTR
P14	F	35	S‐EEG	84 (46)	A^a^,H,E^a^,P^a^	A^a^,H^a^,P^a^	10 (6)	16 (12)	Focal epileptogenic MTR
P15	M	57	S‐EEG	70 (28)	A^a^,H^a^,P^a^	A,H	7 (4)	6 (0)	Focal epileptogenic MTR
P16	M	40	S‐EEG	72 (35)	A,H^a^,E^a^,P	None	13 (4)	0 (0)	Non‐epileptogenic MTR
P17	F	32	S‐EEG	74 (44)	A^a^,H^a^	A,H^a^	6 (6)	5 (2)	Non‐epileptogenic MTR
P18	F	53	S‐EEG	66 (52)	A^a^,H^a^	A^a^,H^a^	9 (8)	7 (6)	Multifocal epileptogenic MTR

Abbreviations: A, amygdala; E, entorhinal cortex; F, female; H, hippocampus; M, male; MTR, mesial temporal region; no., number; P, parahippocampal gyrus; S‐EEG, stereoelectroencephalography.

^a^ Stimulated structure.

### 3.1 Evoked potential distribution

For every bilaterally stimulated patient, the ratio of responses observed within and outside the MTR was not significantly different when stimulating the MTR ipsilateral versus contralateral to seizure onset (Fisher's exact tests, *p* > .05) (Table S2). This was also seen when pooling over patient group (chi‐squared tests, *p* > .05) (Table S3). Similarly, when comparing the proportions of possible responses observed from MTR stimulation, only one patient (P2) had a significantly different proportion observed inside MTR ipsilateral versus contralateral to seizure onset (Fisher's exact tests, *p* < .05) (Table S2), and there were no significant differences at the group level (chi‐squared tests, *p* > .05) (Table S3). However, four patients (P2, P9, P10, P17) had significantly different proportions of possible responses observed outside MTR (Fisher's exact tests, *p* < .05) (Table S2), and the focal‐epileptogenic MTR group had a greater proportion compared to the other patient groups (chi‐squared tests, *p* < .05) (Table S3). While these differences may be attributed to heterogeneous spatial sampling, altogether these results largely indicate that the relative distribution of effective connections produced from stimulating MTR is independent of the epileptogenicity of the MTR.

### 3.2 Evoked potential strength

**FIGURE 2:**
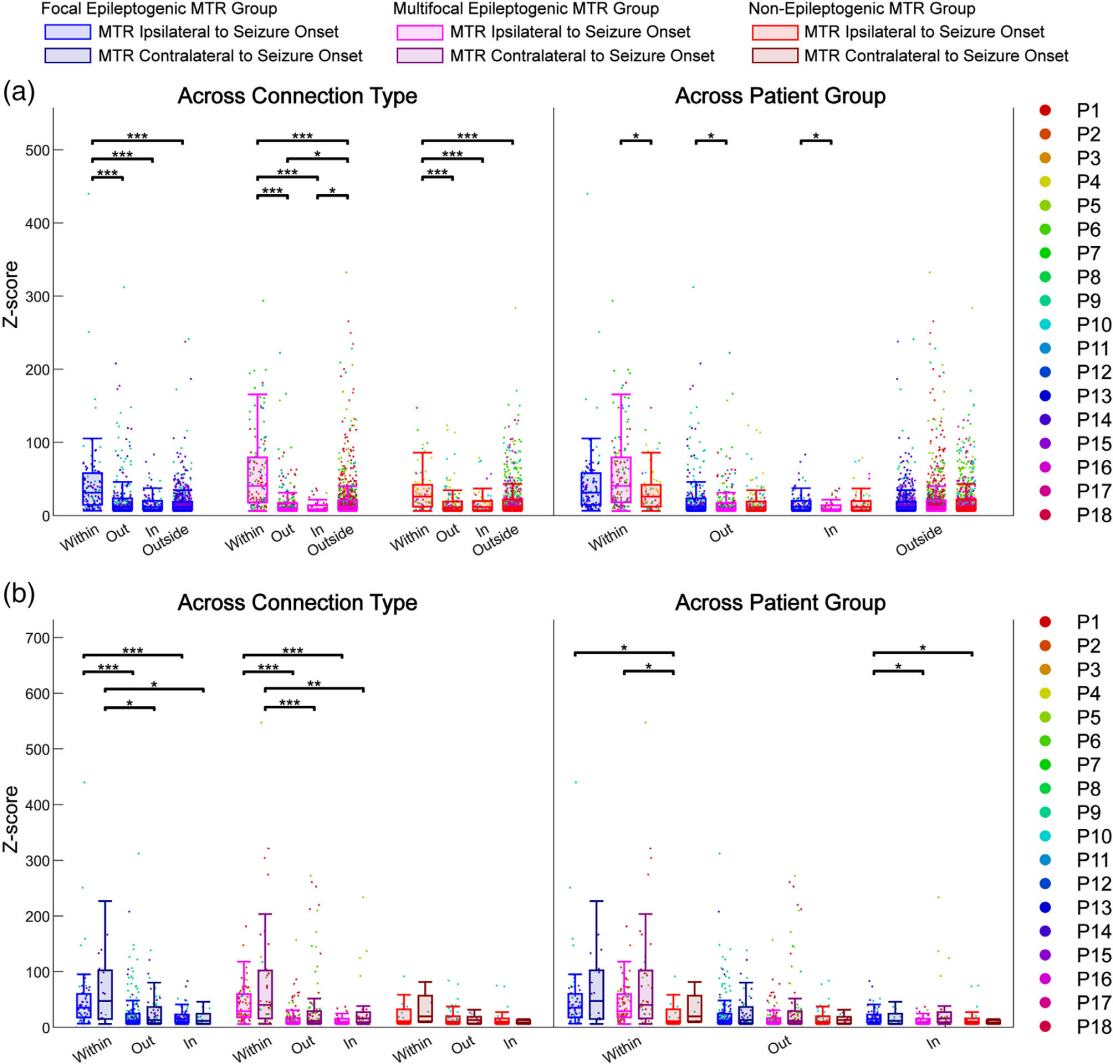
*Z*‐Score comparisons of effective connectivity subnetworks. The *Z*‐scores of significant connections in subnetworks *within*, *out*, *in*, and *outside* relative to MTR are pooled over each patient group [focal epileptogenic MTR (*n* = 4), multifocal epileptogenic MTR (*n* = 9), non‐epileptogenic MTR (*n* = 5)] and compared using grouped Kruskal‐Wallis tests for effects of connection type and patient group, followed by *post hoc* Dunn's tests for pairwise comparisons. (a) Analysis using singular MTR ipsilateral to seizure onset zone across all patients (*n* = 18) showing comparisons across connection type and across patient group. (b) Analysis using MTR ipsilateral and contralateral to seizure onset zone in bilaterally stimulated patients (*n* = 10) showing comparisons across connection type and across patient group. *p < .05; **p < .01; ***p < .001. MTR, mesial temporal region

Among each patient group, median *Z*‐score differed significantly across connection type (Kruskal‐Wallis tests, *p* < .001), and connections within the MTR of each group had greater median *Z*‐scores than that of the other connection types (Dunn's tests, *p* < .001) (Figure 2a, Table S4). This is also seen in contralateral non‐epileptogenic MTR (Dunn's tests, *p* < .05) (Figure 2b, Table S4). Since the relative strength of the effective connections within every observed MTR persists regardless of epileptogenicity, these responses may be indicative of the high physiological connectivity between mesial temporal structures.

There was also a significant difference in median *Z*‐scores across patient groups for *within* [*H*(2) = 10.72, *p* = .0188], *out* [*H*(2) = 6.91, *p* = .0421], and *in* [*H*(2) = 7.38, *p* = .0421] connection types. Specifically, connections within multifocal epileptogenic MTR have greater median *Z*‐scores than those within the non‐epileptogenic MTR (*z* = −3.18, *p* = .0133), and median *Z*‐scores of connections out of (*z* = −2.68, *p* = .0262) and into (*z* = −2.71, *p* = .0262) focal epileptogenic MTR were greater than those of multifocal epileptogenic MTR (Figure 2a, Table S4). In the bilateral analysis, median *Z*‐scores differed across patient group only for connections within [*H*(2) = 9.77, *p* = .0321] and into [*H*(2) = 9.07, *p* = .0321] the MTR ipsilateral to the seizure onset zone (Figure 2b). Connections within focal and multifocal epileptogenic MTR had greater median *Z*‐scores compared to those within non‐epileptogenic MTR (*z* = −3.04, *p* = .0115; *z* = −2.89, *p* = .0115), and connections into focal epileptogenic MTR had greater median *Z*‐scores than those in both other groups (*z* = −2.54, *p* = .0212; *z* = −2.26, *p* = .0357) (Figure 2b, Table S4). Since no significant differences were observed across the contralateral non‐epileptogenic MTR, this may suggest that the variance in *Z*‐scores across the MTR ipsilateral to seizure onset was due to the varying epileptogenicity across patient groups.

### 3.3 Evoked potential weighted density

**FIGURE 3:**
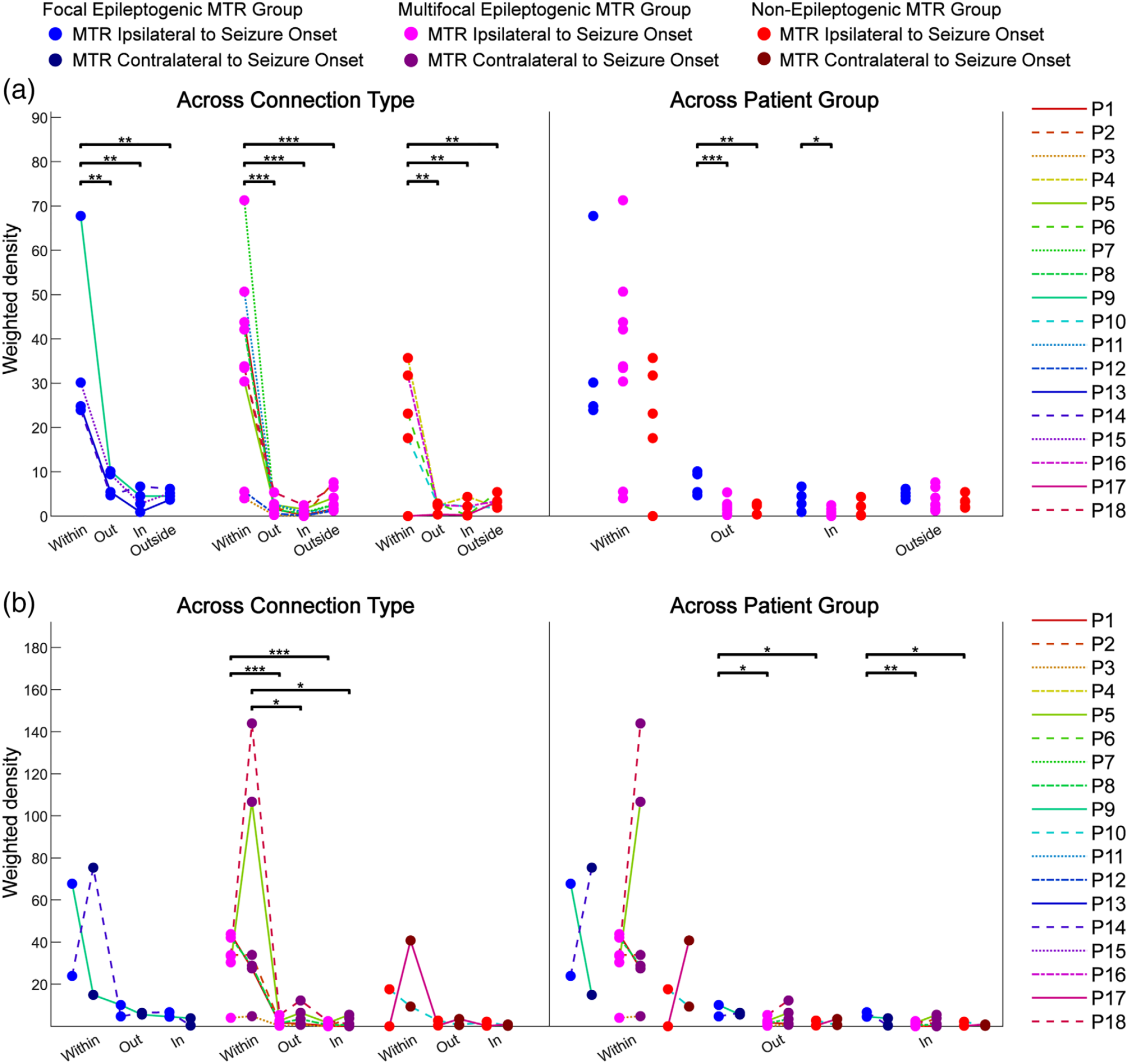
Weighted density comparisons of effective connectivity subnetworks. The weighted densities (sum of significant connections' *Z*‐scores divided by total possible connections) of effective connectivity subnetworks *within*, *out*, *in*, and *outside* relative to MTR are calculated for each patient and fit with linear mixed effects models to compare effects of connection type and patient group, followed by *post hoc* tests for pairwise comparisons. (a) Analysis using singular MTR ipsilateral to seizure onset zone across all patients (*n* = 18) showing comparisons across connection type and across patient group. (b) Analysis using MTR ipsilateral and contralateral to seizure onset zone in bilaterally stimulated patients (*n* = 10) showing comparisons across connection type and across patient group. *p < .05; **p < .01; ***p < .001. MTR, mesial temporal region

Similar to the *Z*‐score results, there was a significant difference in weighted density across connection type within each patient group (LRT, *p <* .001), and connections within the MTR had significantly greater weighted densities than all other connection types for each patient group (*t*‐tests, *p <* .01) (Figure 3a, Table S4). This is also true of the contralateral non‐epileptogenic MTR (*t*‐tests, *p <* .05) (Figure 3b, Table S4), which fits with the *Z*‐score results of a highly dense and strong effective network within MTR that persists across epileptogenicity.

The weighted densities of connections out of focal epileptogenic MTR were significantly greater than that of connections out of both multifocal and non‐epileptogenic MTR [*t*(15) = −5.13, *p* < .0007; *t*(15) = −4.55, *p* = .0011], and the weighted densities of connections into the focal epileptogenic MTR were greater than those into multifocal epileptogenic MTR [*t*(15) = −3.24, *p* = .0109] (Figure 3a, Table S4). In the bilateral analysis, differences across groups were limited to connections out of and into the ipsilateral MTR (Figure 3b). *Out* and *in* connections for focal epileptogenic MTR had greater weighted densities than that of multifocal and non‐epileptogenic MTR [*out* t(7) = −2.96, *p* = .0420 and *t*(7) = −2.73, *p* = .0442; *in t*(7) = −5.12, *p* = .0082 and *t*(7) = −3.83, *p* = .0194] (Figure 3a, Table S4). The densities of the contralateral non‐epileptogenic MTR did not significantly differ across groups, again suggesting that the differences across group in the ipsilateral case were due to the epileptogenicity of the MTR.

### 3.4 Pooled network centrality

**FIGURE 4:**
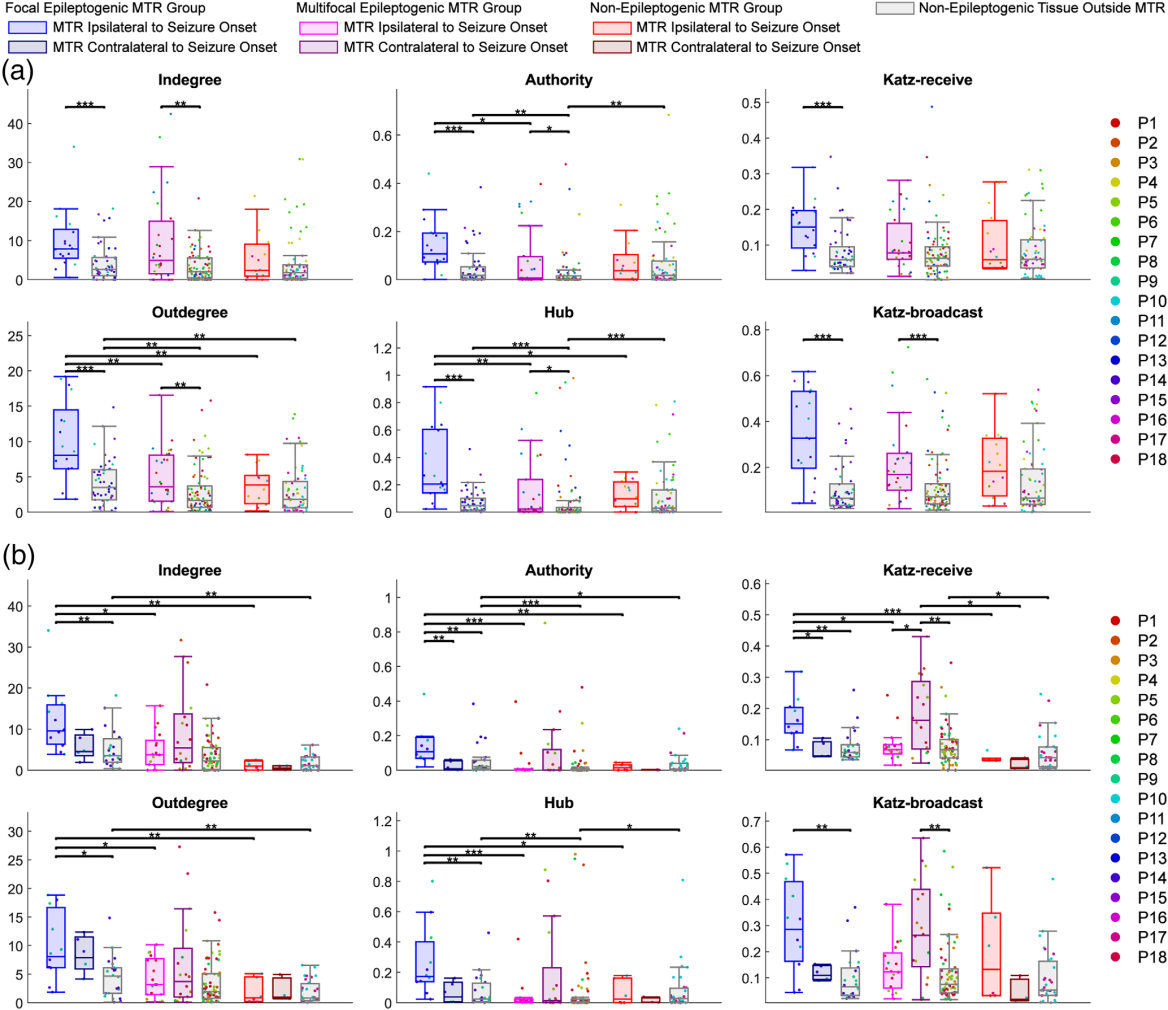
Graph centrality measures of nodes pooled within patient group. The centralities of every node within each location group (inside MTR ipsilateral to seizure onset, inside MTR contralateral to seizure onset, outside MTR in non‐epileptogenic tissue) are pooled within each patient group [focal epileptogenic MTR (*n* = 4), multifocal epileptogenic MTR (*n* = 9), non‐epileptogenic MTR (*n* = 5)] and compared using grouped Kruskal‐Wallis tests for effects of location and patient group, followed by *post hoc* Dunn's tests for pairwise comparisons. (a) Analysis using singular MTR ipsilateral to seizure onset zone across all patients (*n* = 18). (b) Analysis using MTR ipsilateral and contralateral to seizure onset zone in bilaterally stimulated patients (*n* = 10). *p < .05; **p < .01; ***p < .001. MTR, mesial temporal region

A significant difference in the median pooled centrality of nodes within versus outside mesial temporal structures was observed in the focal epileptogenic MTR group for every centrality measure and in the multifocal epileptogenic MTR group for every measure except Katz‐receive (Kruskal‐Wallis tests, *p <* .05) (Figure 4a, Table S4). In each case, the median centrality of electrodes within the MTR was significantly greater than those outside (Dunn's tests, *p <* .05) and the difference was greater for the focal group than for multifocal group. The median centrality of electrodes within the MTR differed significantly across patient group for outdegree, authority, and hub (Kruskal‐Wallis tests, *p <* .05). The median centrality of electrodes within the focal epileptogenic MTR was significantly greater than that within the multifocal and non‐epileptogenic MTR (outdegree *z = −*2.75, *p* = .0088 and *z = −*3.20, *p* = .0084; authority *z = −*2.66, *p* = .0154 and *z = −*1.97, *p* = .0728; hub *z = −*3.09, *p* = .0039 and *z = −*2.13, *p* = .0498) (Figure 4a, Table S4).

When the contralateral non‐epileptogenic MTR was included for the bilaterally stimulated patients, there was still a significant effect of location on centrality for the focal epileptogenic MTR group in every centrality measure (Kruskal‐Wallis tests, *p* < .05) (Figure 4b, Table S4). In each measure, the median centrality within the ipsilateral MTR was still greater than the median centrality outside, while only the median authority and Katz‐receive centrality within the focal epileptogenic MTR was significantly greater than the contralateral MTR (Dunn's tests, *p* < .05). Unexpectedly, in Katz‐receive and Katz‐broadcast centrality, the contralateral non‐epileptogenic MTR within the multifocal patient group was greater than the outside non‐epileptogenic electrodes. There was a significant difference in median centrality across patient group among the MTR ipsilateral to seizure onset for every centrality except Katz‐broadcast (Kruskal‐Wallis tests, *p* < .01), and the electrodes within the focal epileptogenic MTR had greater median centrality than those in both the multifocal and non‐epileptogenic MTR ipsilateral to seizure onset (Dunn's tests, *p* < .01) (Figure 4b, Table S4).

### 3.5 Average network centrality

**FIGURE 5:**
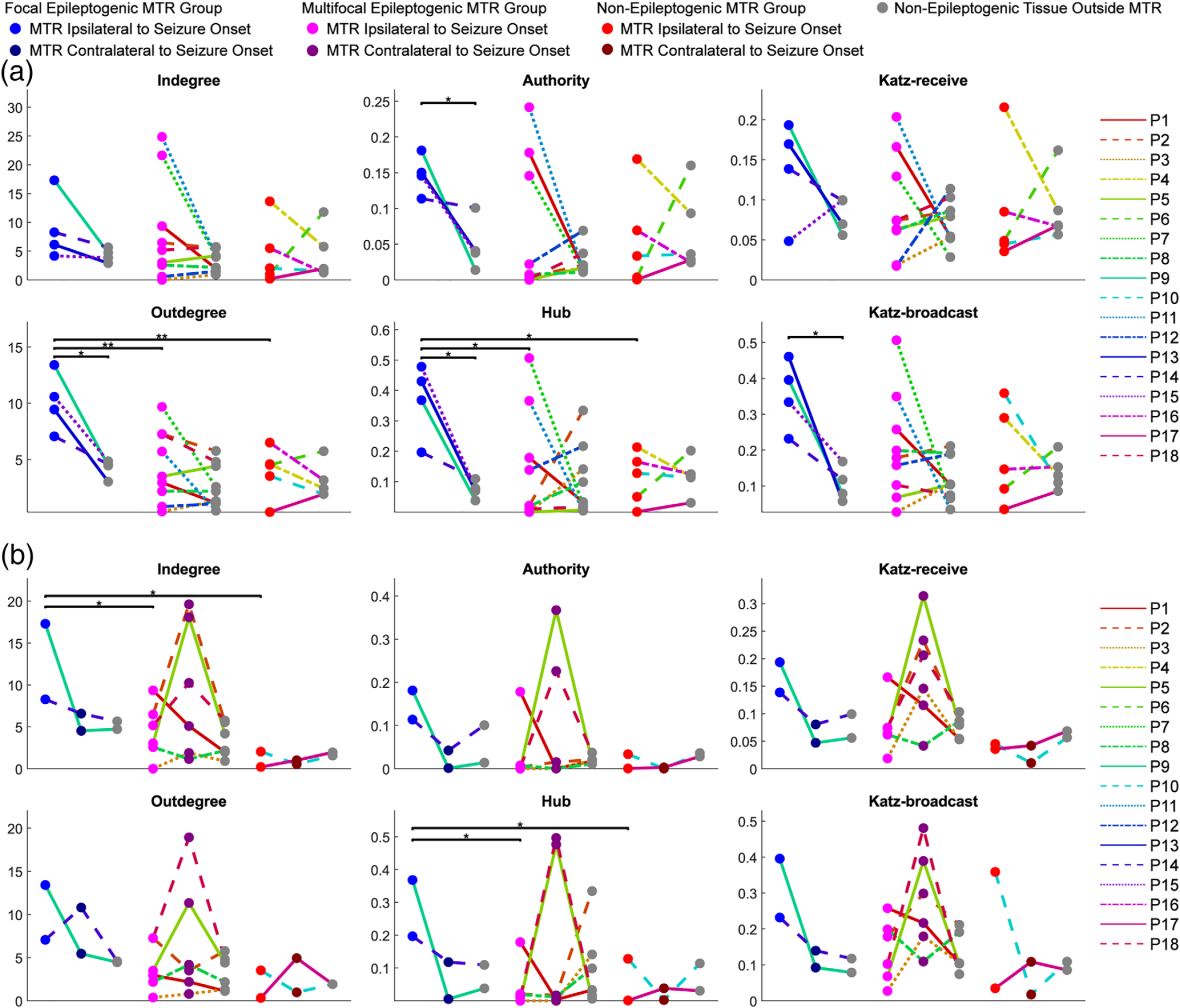
Graph centrality measures of nodes averaged for each patient. The centralities of nodes are averaged for each patient by location (inside MTR ipsilateral to seizure onset, inside MTR contralateral to seizure onset, outside MTR in non‐epileptogenic tissue) and fit with linear mixed effects models to compare effects of location and patient group, followed by *post hoc* tests for pairwise comparisons. (a) Analysis using singular MTR ipsilateral to seizure onset zone across all patients (*n* = 18). (b) Analysis using MTR ipsilateral and contralateral to seizure onset zone in bilaterally stimulated patients (*n* = 10). *p < .05; **p < .01. MTR, mesial temporal region

There was a significant difference in the average centrality of nodes across location for every out‐related measure plus authority within the focal epileptogenic group (LRT, *p* < .05), and the average centrality within focal epileptogenic MTR was greater than the centrality of the non‐epileptogenic tissue outside [outdegree *t*(3) = −4.43, *p =* .0214; authority *t*(3) = −4.31, *p =* .0230; hub *t*(3) = −4.66, *p =* .0187; Katz‐broadcast *t*(3) = −4.59, *p =* .0194] (Figure 5a, Table S4). Centrality within MTR was significantly different across patient group for outdegree and hub centrality (LRT, *p* < .05). Accordingly, average centrality within focal epileptogenic MTR was significantly greater than that within multifocal and non‐epileptogenic MTR for both outdegree and hub [outdegree *t*(15) = −3.30, *p* = .0085 and *t*(15) = −3.23, *p* = .0085; hub *t*(15) = −2.52, *p* = .0355 and *t*(15) = −2.52, *p* = .0355].

When the patients were limited to those bilaterally stimulated, the results were slightly different. Average centrality significantly differed across patient group in indegree, hub, and Katz‐receive centrality for MTR ipsilateral to seizure onset only [indegree *LRT*(2) = 9.45, *p* = .0266; Katz‐receive *LRT*(2) = 8.39, *p* = .0452; hub *LRT*(2) = 10.82, *p* = .0133] (Figure 5b, Table S4). The focal epileptogenic MTR had significantly greater average centrality than multifocal and non‐epileptogenic MTR for indegree [*t*(7) = −2.76, *p* = .0421; *t*(7) = −3.15, *p* = .0421] and hub [*t*(7) = −3.66, *p* = .0243; *t*(7) = −2.66, *p* = .0484]. While similar trends were seen for other centrality measures, they did not reach statistical significance. Katz‐receive and Katz‐broadcast centrality within each group were significantly different across location (LRT, *p* < .05), but *post hoc* analysis did not result in significant pairwise differences.

### 3.6 Outcome prediction

To see how these connectivity metrics of the effective network could offer insight into the role the MTR plays in seizure onset, we looked at outcomes of patients in the multifocal and non‐epileptogenic MTR groups whose centrality measures most resembled those seen in the focal epileptogenic MTR group. For the patients in the multifocal group, this included P1, P2, P7, and P11. While P7 and P11 have not had surgery yet, P1 had an amygdalohippocampectomy with a one year outcome of Engel Class II and P2 had an amygdalohippocampectomy and temporal lobectomy with a one year outcome of Engel Class I, suggesting that the MTR was more important within the multifocal SOZ and larger epileptic network. The patient in the non‐epileptogenic group whose centrality most resembled this pattern is P4, and while the SOZ did not include electrodes within mesial temporal structures, this patient's surgery ablated the amygdala in addition to the initial clinically‐labeled SOZ in temporal pole.

## 4 DISCUSSION

In summary, our data indicate that there are at least three different seizure networks that can be defined by the strength of inward and outward connectivity with respect to the mesial temporal region: focal, multifocal, and non‐epileptogenic networks. Epileptogenicity appears to be associated with stronger and denser inward and outward connectivity. Specifically, an MTR is that is involved in focal seizure onset can better synchronize epileptiform discharges and has stronger outward influence on the network compared to an MTR that is not involved in seizure onset or included in a broader seizure onset. We applied graph theoretical analyses to demonstrate that the epileptogenic MTR that is involved in focal seizure onset has more effective connectivity with the rest of the brain when compared to non‐epileptogenic MTRs, potentially indicating an elevated propagative influence over the network. Overall, our findings suggest that the focal epileptogenic MTR plays a critical role in ictogenesis and seizure propagation primarily due to the density of its connections with the remainder of the brain, with increased susceptibility to network perturbations and widespread influence over the effective network.

Although the use of SPES to understand large scale epileptogenic networks is limited, previous studies investigating evoked responses in mesial temporal structures have revealed robust effective connectivity between these structures and functionally related regions in temporal neocortex and limbic structures (Catenoix, Magnin, Mauguière, & Ryvlin, 2011; David et al., 2013; Enatsu et al., 2015; Lacruz, García Seoane, Valentin, Selway, & Alarcón, 2007; Lacuey et al., 2014; Mégevand et al., 2017; Novitskaya, Dümpelmann, Vlachos, Reinacher, & Schulze‐Bonhage, 2020; Wilson, Isokawa, Babb, & Crandall, 1990). We similarly observed that the evoked potentials within the mesial temporal structures (amygdala, hippocampus, entorhinal cortex, and parahippocampal gyrus) consistently showed greater magnitude and density compared to connections throughout the rest of the brain, regardless of the epileptogenicity of the MTR. This is consistent with our understanding that these structures are part of a larger limbic circuit; indeed, coherent function of MTR is necessary for tasks such as memory formation and olfactory processing (West & Doty, 1995).

Matsumoto, Kunieda, and Nair (2017) proposed that the epileptic condition may increase the strength of functional connections without altering the distribution of these connections. Several studies support similar conclusions; greater evoked responses can be produced from stimulating within the seizure onset zone (Enatsu, Piao, et al., 2012) and higher amplitude responses are evoked within seizure onset zone from stimulation outside (Enatsu, Jin, et al., 2012; Iwasaki et al., 2010; Parker et al., 2018). Accordingly, we found that the response amplitude and weighted density of connections within focal and multifocal epileptogenic MTR was generally greater than that within non‐epileptogenic MTR. However, we observed that the relative distribution of responses within and outside the MTR did not vary significantly when comparing stimulation of the epileptogenic MTR to the contralateral non‐epileptogenic MTR within the same patient. This is congruent with previous studies that have shown similar connection distributions between epileptogenic and non‐epileptogenic regions (Lacruz et al., 2007; Wilson et al., 1990). While we also noted that epileptogenicity may alter the proportion of possible responses outside the MTR, future analysis incorporating spatially homogenous sampling is needed to confirm this preliminary finding.

There is now widespread acceptance that focal epilepsy is not limited to a small region of the brain, but is a phenomenon rising from aberrant large‐scale connectivity. Spencer (2002) proposed in her seminal paper the following concept: “vulnerability to seizure activity in any one part of the network is influenced by activity everywhere else in the network, and that the network as a whole is responsible for the clinical and electrographic phenomena that we associate with human seizures”. Based on the graph theoretical analysis of network centrality, we conclude that the MTR is both a site of most relevant propagation of activity while also acting, to a lesser degree, as a receiver of activity within the network. While we did observe larger magnitude and density of responses within focal epileptogenic MTR in some cases, we also noted that the centrality measures that quantify the ability to receive activity (indegree, authority, Katz‐receive) were not as significantly different across different levels of epileptogenicity. However, for each centrality measure that quantified the ability to propagate activity (outdegree, hub, Katz‐broadcast), only the focal epileptogenic MTR had significantly greater average centrality than the outside non‐epileptogenic tissue. The pooled analysis showed a similar difference for multifocal epileptogenic MTR but at a lesser magnitude. This suggests that epileptogenicity of the MTR is associated with an elevated propagative influence over the effective network that can increase as the seizure onset zone is more localized to mesial temporal structures. The increased role in both receiving and propagating activity makes the epileptogenic MTR an unstable node in the network, because not only can it respond to perturbations in the network due its greater excitability, it is also more capable of propagating this activity throughout the brain. Our finding is consistent with previous studies that have shown nodes within the seizure onset zone to have both greater indegree and outdegree (Parker et al., 2018; van Blooijs et al., 2018) or as being highly bidirectionally connected, acting as a receiver and activator of evoked potentials (Boido et al., 2014).

### 4.1 Strengths and limitations

This study is unique in comparing epileptogenic MTR not only to non‐epileptogenic MTR, but also to the contralateral non‐epileptogenic MTR within the same patient. This direct comparison offered additional insight supporting the proposal in Matsumoto et al. (2017) that the relative distributions of effective connections is not affected by epileptogenicity. However, we did not observe many significant differences in the connectivity metrics when directly comparing epileptogenic MTR to the contralateral non‐epileptogenic MTR, perhaps due to limited spatial sampling in contralateral non‐epileptogenic MTR structures. Yet, we did see that the significant differences in response magnitude, density, and centrality across patient groups in the MTR ipsilateral to seizure onset were almost never observed in the contralateral non‐epileptogenic MTR. This indicates that the differences we did observe were due to the varying epileptogenicity of the ipsilateral MTR, rather than another confounding variable across patient groups.

For this study, we defined epileptogenic electrodes as those within the clinically defined SOZ. While additional clinically annotated sites of early seizure propagation or irritative zones may be involved in a greater seizure network, they were not deemed responsible for seizure onset and therefore considered non‐epileptogenic. However, some studies have found that electrodes that show early propagation of seizure activity can be more excitable to SPES than the rest of the network (Lega et al., 2015; Parker et al., 2018). This may have contributed to the unexpectedly high centrality of some of the contralateral non‐epileptogenic MTR in the multifocal group, as each patient in this group had electrodes within contralateral non‐epileptogenic MTR classified as early propagation or irritative sites. Additional unexpected significant differences in centrality of non‐epileptogenic regions across patient groups may be similarly explained or due to differences in electrode coverage and stimulation across patients.

One potential drawback of this study is the grouping of amygdala, hippocampus, entorhinal cortex, and parahippocampal gyrus as one collective mesial temporal region of interest. While these structures were chosen in part due to consistent electrode coverage, they were grouped in this way primarily because of their joint involvement in seizure onset across patients. These structures rank among the highest epileptogenic involvement in mTLE cases, with multiple structures involved per patient, indicative of a network involvement rather than isolation to singular structures (Bartolomei, Chauvel, & Wendling, 2008). Additionally, these structures are robustly connected through evoked responses between each other (Catenoix et al., 2011; Enatsu et al., 2015; Wilson et al., 1990) and to cerebral cortex (Mégevand et al., 2017). However, we recognize that separate analysis of each structure's connectivity may provide additional insight to account for patient variability in structural epileptogenicity, differences in physiological connectivity, and asymmetry of bidirectional effective connections (Mégevand et al., 2017; Novitskaya et al., 2020). This level of detail would require more extensive sampling of each structure within and across patients and is outside the scope of this study, but a future study with a larger patient pool could investigate these differences.

### 4.2 Directions and clinical applications

While this study focused on effective network properties from evoked potentials produced by SPES, higher frequency activity evoked by SPES can also be used to characterize the electrophysiological connectivity between stimulated brain regions. These evoked spectral responses can provide measures of functional connectivity (Crowther et al., 2019; Gkogkidis et al., 2017) and can be modulated by repetitive cortical stimulation (Keller et al., 2018; Huang et al., 2019) or states of wakefulness and sleep (Usami et al., 2019). Further, electrodes with early responses in the ripple and fast ripple frequency range are indicative of epileptogenicity (Mălîia et al., 2017; Mouthaan et al., 2016; van 't Klooster et al., 2011). Matsumoto et al. (2017) indicates that this increase in high frequency activity may be even greater mTLE cases. A future study examining the evoked spectral responses in epileptogenic and non‐epileptogenic MTR could provide additional insight to how epileptogenicity can modulate the network influence of nodes within the MTR.

While no predictive model is presented here, the unique effective network properties of focal epileptogenic MTR could be useful for assessing the importance of the involvement of the MTR in ictogenesis and seizure propagation in patients with mTLE. In future studies, we aim to study the role of the observed effective network properties as an evaluative tool to improve surgical outcomes.

## 5 CONCLUSION

With epilepsy increasingly viewed as a network disease, a deeper understanding of the underlying electrophysiological interactions of brain regions and the mechanisms responsible for seizure generation and propagation through this network is crucial to improving the management of epilepsy and success of surgical outcomes. Here, the combination of graph theoretical analyses with SPES offers a novel method of characterizing the causal influence of focal epileptogenic brain regions within larger electrophysiological networks. By comparing the effective network properties of mesial temporal structures with varying degrees of epileptogenicity within and across patients, we demonstrate that the MTR is a strong, reciprocally connected subnetwork, with many bidirectional connections within and to surrounding network. We infer that the epileptogenic MTR is an important site of origination for seizure propagation while also acting, to a lesser degree, as a receiver of activity within the epileptogenic network. This elevated propagative influence over the effective network increases as the SOZ is more localized to mesial temporal structures. Our findings provide concrete evidence of network effects of mTLE and provide insights into how current and future therapeutic approaches might be optimized to address these effects.

## CONFLICT OF INTEREST

The authors declare no potential conflict of interest.

## Supporting information

**Appendix S1**: Supporting Information.

## Data Availability

Deidentified data that support the findings of this study are available from the corresponding author upon reasonable request.
