# Supplementary material for: Graph theoretical analysis of evoked potentials shows network influence of epileptogenic mesial temporal region
Source: Hum Brain Mapp. 2021 Jun 24;42(13):4173–86. doi: 10.1002/hbm.25418 (PMC8356982; doi:10.1002/hbm.25418)
Supplement: Supplementary file 1 — Appendix S1: Supporting Information. [file HBM-42-4173-s001.docx]

Supplementary Table 1: Stimulation current intensities.

| Patient No. | Mean (Variance) of Current Intensity for Sites Within MTR [mA] | | Mean (Variance) of Current Intensity for Sites Outside MTR [mA] | Mean (Variance) of Current Intensity for Sites Within SOZ [mA] | Mean (Variance) of Current Intensity for Sites Outside SOZ [mA] |
| --- | --- | --- | --- | --- | --- |
|  | Left | Right |  |  |  |
| P1 | 5 (0) | 4.333 (1.333) | 3.818 (1.764) | 4.6 (0.8) | 3.818 (1.764) |
| P2 | 4 (0) | 4 (0) | 9.143 (5.143) | 7 (12) | 6.667 (10) |
| P3 | 2.65 (0.045) | 1.65 (0.79) | 3.006 (1.007) | 2.3875 (0.387) | 2.921 (1.4882) |
| P4 | 3.6 (0.178) | None | 3.933 (0.444) | 3.75 (0.125) | 3.861 (0.423) |
| P5 | 2.333 (0.333) | 1.5 (0.25) | 5.658 (3.501) | 4.4375 (5.389) | 4.912 (5.632) |
| P6 | 4 (0) | None | 4.029 (0.015) | 4 (0) | 4.033 (0.017) |
| P7 | 4.5 (0) | None | 4.5 (0) | 4.5 (0) | 4.5 (0) |
| P8 | 5.5 (0) | 5.5 (0) | 5.5 (0) | 5.5 (0) | 5.5 (0) |
| P9 | 6 (0) | 6 (0) | 6 (0) | 6 (0) | 6 (0) |
| P10 | 5 (0) | 5 (0) | 5 (0) | 5 (0) | 5 (0) |
| P11 | None | 5 (0) | 4.6 (0.8) | 5 (0) | 4.714 (0.571) |
| P12 | None | 6 (0) | 6 (0) | 6 (0) | 6 (0) |
| P13 | 6 (0) | None | 6 (0) | 6 (0) | 6 (0) |
| P14 | 6 (0) | 4.167 (4.167) | 5.929 (0.071) | 4.5 (3.429) | 6 (0) |
| P15 | 5 (0) | None | 5 (0) | 5 (0) | 5 (0) |
| P16 | 5 (0) | None | 5 (0) | 5 (0) | 5 (0) |
| P17 | 6 (0) | 6 (0) | 5.778 (0.418) | 6 (0) | 5.75 (0.467) |
| P18 | 5 (0) | 5 (0) | 5 (0) | 5 (0) | 5 (0) |

No., Number; MTR, mesial temporal region; SOZ, seizure onset zone

Supplementary Table 2: Mesial temporal region evoked potential distribution comparisons.

| Patient No. | Stimulation Location | No. of Responses | | | | Fisher’s exact test *P*-value | | |
| --- | --- | --- | --- | --- | --- | --- | --- | --- |
|  |  | Observed Within MTR | Possible Within MTR | Observed Outside MTR | Possible Outside MTR | Ratio of Response Within to Outside | Proportion of Possible Response Within | Proportion of Possible Response Outside |
| 1 | Ipsilateral | 8 | 1 | 24 | 218 | 0.4726 | 0.5500 | 0.0901 |
|  | Contralateral | 5 | 2 | 8 | 153 |  |  |  |
| 2 | Ipsilateral | 43 | 10 | 31 | 194 | 0.2628 | 0.0035 | 0.0188 |
|  | Contralateral | 12 | 14 | 15 | 209 |  |  |  |
| 3 | Ipsilateral | 2 | 8 | 6 | 209 | 1 | 1 | 1 |
|  | Contralateral | 5 | 14 | 13 | 418 |  |  |  |
| 5 | Ipsilateral | 6 | 3 | 21 | 159 | 0.2621 | 0.0737 | 0.8670 |
|  | Contralateral | 11 | 0 | 19 | 161 |  |  |  |
| 8 | Ipsilateral | 3 | 0 | 20 | 135 | 1 | 0.4643 | 0.8657 |
|  | Contralateral | 3 | 3 | 19 | 137 |  |  |  |
| 9 | Ipsilateral | 23 | 4 | 123 | 219 | 1 | 1 | 0.0048 |
|  | Contralateral | 5 | 1 | 31 | 107 |  |  |  |
| 10 | Ipsilateral | 16 | 5 | 41 | 238 | 0.7194 | 0.1055 | 0.0001 |
|  | Contralateral | 4 | 6 | 7 | 179 |  |  |  |
| 14 | Ipsilateral | 40 | 19 | 72 | 233 | 0.7167 | 0.0539 | 0.7238 |
|  | Contralateral | 15 | 1 | 33 | 118 |  |  |  |
| 17 | Ipsilateral | 0 | 6 | 4 | 122 | 1 | 0.2500 | 0.0001 |
|  | Contralateral | 1 | 1 | 11 | 31 |  |  |  |
| 18 | Ipsilateral | 13 | 4 | 42 | 113 | 1 | 0.2631 | 1 |
|  | Contralateral | 31 | 85 | 31 | 85 |  |  |  |

No., Number; MTR, Mesial temporal region.

Supplementary Table 3: Mesial temporal region evoked potential distribution pooled comparisons.

| Patient Group | Stimulation Location | No. of Responses | | | | Chi-squared test *P*-value | | |
| --- | --- | --- | --- | --- | --- | --- | --- | --- |
|  |  | Observed Within MTR | Possible Within MTR | Observed Outside MTR | Possible Outside MTR | Ratio of Response Within to Outside | Proportion of Possible Response Within | Proportion of Possible Response Outside |
| Focal epileptogenic MTR | Ipsilateral | 63 | 23 | 195 | 452 | 0.9100 | 0.0798 | 0.0116 |
|  | Contralateral | 20 | 2 | 64 | 225 |  |  |  |
| Multifocal epileptogenic MTR | Ipsilateral | 75 | 26 | 144 | 1028 | 0.3924 | 0.0194 | 0.0011 |
|  | Contralateral | 45 | 33 | 105 | 1163 |  |  |  |
| Non-epileptogenic MTR | Ipsilateral | 16 | 11 | 45 | 360 | 0.6717 | 0.3091 | 0.1944 |
|  | Contralateral | 5 | 7 | 18 | 210 |  |  |  |

MTR, Mesial temporal region.

Supplementary Table 4: Detailed test statistics and *P*-values of Z-score, weighted density, and centrality comparisons referenced in figures and text.

| Figure | Statistical analysis | Test Statistic | *P*-value (FDR corrected) |
| --- | --- | --- | --- |
| 2A (left) | Focal, Kruskal-Wallis test | *H*(3) = 83.97 | <0.0001 |
| 2A (left) | Multifocal, Kruskal-Wallis test | *H*(3) = 150.22 | <0.0001 |
| 2A (left) | Non, Kruskal-Wallis test | *H*(3) = 31.33 | <0.0001 |
| 2A (left) | Focal, within vs out, Dunn’s test | *z* = -7.45 | 0.0001 |
| 2A (left) | Focal, within vs in, Dunn’s test | *z* = -6.39 | <0.0001 |
| 2A (left) | Focal, within vs outside, Dunn’s test | *z* = -9.15 | <0.0001 |
| 2A (left) | Multifocal, within vs out, Dunn’s test | *z* = -11.1 | <0.0001 |
| 2A (left) | Multifocal, within vs in, Dunn’s test | *z* = -8.26 | <0.0001 |
| 2A (left) | Multifocal, within vs outside, Dunn’s test | *z* = -11.1 | <0.0001 |
| 2A (left) | Multifocal, out vs in, Dunn’s test | *z* = 2.43 | 0.0249 |
| 2A (left) | Multifocal, out vs outside, Dunn’s test | *z* = 2.66 | 0.0139 |
| 2A (left) | Non, within vs out, Dunn’s test | *z* = -5.01 | <0.0001 |
| 2A (left) | Non, within vs in, Dunn’s test | *z* = -4.52 | <0.0001 |
| 2A (left) | Non, within vs outside, Dunn’s test | *z* = -5.34 | <0.0001 |
| 2A (right) | Within, Kruskal-Wallis test | *H*(2) = 10.72 | 0.0188 |
| 2A (right) | Out, Kruskal-Wallis test | *H*(2) = 6.91 | 0.0421 |
| 2A (right) | In, Kruskal-Wallis test | *H*(2) = 7.38 | 0.0421 |
| 2A (right) | Within, multifocal vs non, Dunn’s test | *z* = -3.18 | 0.0133 |
| 2A (right) | Out, focal vs multifocal, Dunn’s test | *z* = -2.62 | 0.0262 |
| 2A (right) | In, focal vs multifocal, Dunn’s test | *z* = -2.71 | 0.0262 |
| 2B (left) | Focal, ipsilateral, Kruskal-Wallis test | *H*(2) = 40.38 | <0.0001 |
| 2B (left) | Multifocal, ipsilateral, Kruskal-Wallis test | *H*(2) = 67.34 | <0.0001 |
| 2B (left) | Focal, contralateral, Kruskal-Wallis test | *H*(2) = 9.09 | 0.0159 |
| 2B (left) | Multifocal, contralateral, Kruskal-Wallis test | *H*(2) = 21.48 | <0.0001 |
| 2B (left) | Focal, ipsilateral, within vs out, Dunn’s test | *z* = -6.30 | <0.0001 |
| 2B (left) | Focal, ipsilateral, within vs in, Dunn’s test | *z* = -4.70 | <0.0001 |
| 2B (left) | Focal, contralateral, within vs out, Dunn’s test | *z* = -2.64 | 0.0125 |
| 2B (left) | Focal, contralateral, within vs in, Dunn’s test | *z* = -2.69 | 0.0123 |
| 2B (left) | Multifocal, ipsilateral, within vs out, Dunn’s test | *z* = -7.77 | <0.0001 |
| 2B (left) | Multifocal, ipsilateral, within vs in, Dunn’s test | *z* = -5.87 | <0.0001 |
| 2B (left) | Multifocal, contralateral, within vs out, Dunn’s test | *z* = -4.62 | 0.0001 |
| 2B (left) | Multifocal, contralateral, within vs in, Dunn’s test | *z* = -2.82 | 0.0095 |
| 2B (right) | Within, ipsilateral, Kruskal-Wallis test | *H*(2) = 9.77 | 0.0321 |
| 2B (right) | In, ipsilateral, Kruskal-Wallis test | *H*(2) = 9.07 | 0.0321 |
| 2B (right) | Within, ipsilateral, focal vs non, Dunn’s test | *z* = -3.04 | 0.0115 |
| 2B (right) | Within, ipsilateral, multifocal vs non, Dunn’s test | *z* = -2.89 | 0.0115 |
| 2B (right) | In, ipsilateral, focal vs multifocal, Dunn’s test | *z* = -2.54 | 0.0212 |
| 2B (right) | In, ipsilateral, focal vs non, Dunn’s test | *z* = -2.26 | 0.0357 |
| 3A (left) | Focal, likelihood-ratio test | *LRT*(3) = 18.67 | 0.0003 |
| 3A (left) | Multifocal, likelihood-ratio test | *LRT*(3) = 40.11 | <0.0001 |
| 3A (left) | Non, likelihood-ratio test | *LRT*(3) = 20.21 | 0.0002 |
| 3A (left) | Focal, within vs out, *t-*test | *t*(9) = -4.03 | 0.0059 |
| 3A (left) | Focal, within vs in, *t-*test | *t*(9) = -4.54 | 0.0036 |
| 3A (left) | Focal, within vs outside, *t-*test | *t*(9) = -4.38 | 0.0040 |
| 3A (left) | Multifocal, within vs out, *t-*test | *t*(24) = -6.70 | 0.0001 |
| 3A (left) | Multifocal, within vs in, *t-*test | *t*(24) = -6.98 | 0.0001 |
| 3A (left) | Multifocal, within vs outside, *t-*test | *t*(24) = -6.47 | 0.0001 |
| 3A (left) | Non, within vs out, *t-*test | *t*(12) = -4.53 | 0.0025 |
| 3A (left) | Non, within vs in, *t-*test | *t*(12) = -4.60 | 0.0025 |
| 3A (left) | Non, within vs outside, *t-*test | *t*(12) = -4.28 | 0.0179 |
| 3A (right) | Out, likelihood-ratio test | *LRT*(2) = 19.53 | 0.0002 |
| 3A (right) | In, likelihood-ratio test | *LRT*(2) = 9.60 | 0.0165 |
| 3A (right) | Out, focal vs multifocal, *t-*test | *t*(15) = -5.13 | 0.0007 |
| 3A (right) | Out, focal vs non, *t-*test | *t*(15) = -4.55 | 0.0011 |
| 3A (right) | In, focal vs multifocal, *t-*test | *t*(15) = -3.24 | 0.0109 |
| 3B (left) | Multifocal, ipsilateral, likelihood-ratio test | *LRT*(2) = 26.64 | <0.0001 |
| 3B (left) | Multifocal, contralateral, likelihood-ratio test | *LRT*(2) = 4.83 | 0.0147 |
| 3B (left) | Multifocal, ipsilateral, within vs out, *t-*test | *t*(10) = -6.21 | 0.0003 |
| 3B (left) | Multifocal, ipsilateral, within vs in, *t-*test | *t*(10) = -6.51 | 0.0003 |
| 3B (left) | Multifocal, contralateral, within vs out, *t-*test | *t*(10) = -3.07 | 0.0179 |
| 3B (left) | Multifocal, contralateral, within vs in, *t-*test | *t*(10) = -3.19 | 0.0179 |
| 3B (right) | Out, ipsilateral, likelihood-ratio test | *LRT*(2) = 8.94 | 0.0343 |
| 3B (right) | In, ipsilateral, likelihood-ratio test | *LRT*(2) = 15.81 | 0.0022 |
| 3B (right) | Out, ipsilateral, focal vs multifocal, *t-*test | *t*(7) = -2.96 | 0.0420 |
| 3B (right) | Out, ipsilateral, focal vs non, *t-*test | *t*(7) = -2.73 | 0.0442 |
| 3B (right) | In, ipsilateral, focal vs multifocal, *t-*test | *t*(7) = -5.12 | 0.0082 |
| 3B (right) | In, ipsilateral, focal vs non, *t-*test | *t*(7) = -3.83 | 0.0194 |
| 4A (top, left) | Indegree, focal, Kruskal-Wallis test | *H*(1) = 12.37 | 0.0013 |
| 4A (top, left) | Indegree, multifocal, Kruskal-Wallis test | *H*(1) = 8.24 | 0.0062 |
| 4A (bottom, left) | Outdegree, focal, Kruskal-Wallis test | *H*(1) = 14.69 | 0.0003 |
| 4A (bottom, left) | Outdegree, multifocal, Kruskal-Wallis test | *H*(1) = 6.97 | 0.0125 |
| 4A (top, middle) | Authority, focal, Kruskal-Wallis test | *H*(1) = 15.48 | 0.0003 |
| 4A (top, middle) | Authority, multifocal, Kruskal-Wallis test | *H*(1) = 5.17 | 0.0344 |
| 4A (bottom, middle) | Hub, focal, Kruskal-Wallis test | *H*(1) = 19.95 | <0.0001 |
| 4A (bottom, middle) | Hub, multifocal, Kruskal-Wallis test | *H*(1) = 5.08 | 0.0363 |
| 4A (top, right) | Katz-receive, focal, Kruskal-Wallis test | *H*(1) = 12.77 | 0.0011 |
| 4A (bottom, right) | Katz-broadcast, focal, Kruskal-Wallis test | *H*(1) = 20.08 | <0.0001 |
| 4A (bottom, right) | Katz-broadcast, multifocal, Kruskal-Wallis test | *H*(1) = 11.16 | 0.0013 |
| 4A (top, left) | Indegree, focal, within vs outside, Dunn’s test | *z* = -3.52 | 0.0013 |
| 4A (top, left) | Indegree, multifocal, within vs outside, Dunn’s test | *z* = -2.87 | 0.0062 |
| 4A (bottom, left) | Outdegree, focal, within vs outside, Dunn’s test | *z* = -3.83 | 0.0003 |
| 4A (bottom, left) | Outdegree, multifocal, within vs outside, Dunn’s test | *z* = -2.64 | 0.0125 |
| 4A (top, middle) | Authority, focal, within vs outside, Dunn’s test | *z* = -3.93 | 0.0003 |
| 4A (top, middle) | Authority, multifocal, within vs outside, Dunn’s test | *z* = -2.27 | 0.0344 |
| 4A (bottom, middle) | Hub, focal, within vs outside, Dunn’s test | *z* = -4.47 | <0.0001 |
| 4A (bottom, middle) | Hub, multifocal, within vs outside, Dunn’s test | *z* = -2.25 | 0.0363 |
| 4A (top, right) | Katz-receive, focal, within vs outside, Dunn’s test | *z* = -3.57 | 0.0011 |
| 4A (bottom, right) | Katz-broadcast, focal, within vs outside, Dunn’s test | *z* = -4.48 | <0.0001 |
| 4A (bottom, right) | Katz-broadcast, multifocal, within vs outside, Dunn’s test | *z* = -3.34 | 0.0013 |
| 4A (bottom, left) | Outdegree, within, Kruskal-Wallis test | *H*(2) = 11.73 | 0.0057 |
| 4A (bottom, left) | Outdegree, outside, Kruskal-Wallis test | *H*(2) = 10.08 | 0.0065 |
| 4A (top, middle) | Authority, within, Kruskal-Wallis test | *H*(2) = 7.51 | 0.0284 |
| 4A (top, middle) | Authority, outside, Kruskal-Wallis test | *H*(2) = 16.15 | 0.0006 |
| 4A (bottom, middle) | Hub, within, Kruskal-Wallis test | *H*(2) = 9.88 | 0.0071 |
| 4A (bottom, middle) | Hub, outside, Kruskal-Wallis test | *H*(2) = 20.58 | 0.0001 |
| 4A (bottom, left) | Outdegree, within, focal vs multifocal, Dunn’s test | *z* = -2.75 | 0.0088 |
| 4A (bottom, left) | Outdegree, within, focal vs non, Dunn’s test | *z* = -3.20 | 0.0084 |
| 4A (bottom, left) | Outdegree, outside, focal vs multifocal, Dunn’s test | *z* = -2.86 | 0.0088 |
| 4A (bottom, left) | Outdegree, outside, focal vs non, Dunn’s test | *z* = -2.81 | 0.0088 |
| 4A (top, middle) | Authority, within, focal vs multifocal, Dunn’s test | *z* = -2.66 | 0.0154 |
| 4A (top, middle) | Authority, within, focal vs non, Dunn’s test | *z* = -1.97 | 0.0728 |
| 4A (top, middle) | Authority, outside, focal vs multifocal, Dunn’s test | *z* = -3.47 | 0.0030 |
| 4A (top, middle) | Authority, outside, multifocal vs non, Dunn’s test | *z* = 3.28 | 0.0030 |
| 4A (bottom, middle) | Hub, within, focal vs multifocal, Dunn’s test | *z* = -3.09 | 0.0039 |
| 4A (bottom, middle) | Hub, within, focal vs non, Dunn’s test | *z* = -2.13 | 0.0498 |
| 4A (bottom, middle) | Hub, outside, focal vs multifocal, Dunn’s test | *z* = -4.00 | 0.0004 |
| 4A (bottom, middle) | Hub, outside, multifocal vs non, Dunn’s test | *z* = 3.60 | 0.0009 |
| 4B (top, left) | Indegree, focal, Kruskal-Wallis test | *H*(2) = 9.30 | 0.0287 |
| 4B (bottom, left) | Outdegree, focal, Kruskal-Wallis test | *H*(2) = 10.60 | 0.0140 |
| 4B (top, middle) | Authority, focal, Kruskal-Wallis test | *H*(2) = 11.67 | 0.0087 |
| 4B (bottom, middle) | Hub, focal, Kruskal-Wallis test | *H*(2) = 11.38 | 0.0101 |
| 4B (top, right) | Katz-receive, focal, Kruskal-Wallis test | *H*(2) = 13.14 | 0.0042 |
| 4B (top, right) | Katz-receive, multifocal, Kruskal-Wallis test | *H*(2) = 11.04 | 0.0060 |
| 4B (bottom, right) | Katz-broadcast, focal, Kruskal-Wallis test | *H*(2) = 12.20 | 0.0034 |
| 4B (bottom, right) | Katz-broadcast, multifocal, Kruskal-Wallis test | *H*(2) = 13.15 | 0.0034 |
| 4B (top, left) | Indegree, focal, ipsilateral vs outside, Dunn’s test | *z* = -3.04 | 0.0070 |
| 4B (bottom, left) | Outdegree, focal, ipsilateral vs outside, Dunn’s test | *z* = -2.93 | 0.0103 |
| 4B (top, middle) | Authority, focal, ipsilateral vs contralateral, Dunn’s test | *z* = -2.75 | 0.0089 |
| 4B (top, middle) | Authority, focal, ipsilateral vs outside, Dunn’s test | *z* = -3.07 | 0.0065 |
| 4B (bottom, middle) | Hub, focal, ispi vs outside, Dunn’s test | *z* = -3.31 | 0.0028 |
| 4B (top, right) | Katz-receive, focal, ipsilateral vs contralateral, Dunn’s test | *z* = -2.53 | 0.0215 |
| 4B (top, right) | Katz-receive, focal, ipsilateral vs outside, Dunn’s test | *z* = -3.47 | 0.0030 |
| 4B (top, right) | Katz-receive, multifocal, ipsilateral vs contralateral, Dunn’s test | *z* = 2.45 | 0.0215 |
| 4B (top, right) | Katz-receive, multifocal, contralateral vs outside, Dunn’s test | *z* = -3.29 | 0.0030 |
| 4B (bottom, right) | Katz-broadcast, focal, ipsilateral vs outside, Dunn’s test | *z* = -3.48 | 0.0015 |
| 4B (bottom, right) | Katz-broadcast, multifocal, contralateral vs outside, Dunn’s test | *z* = -3.58 | 0.0015 |
| 4B (top, left) | Indegree, ipsilateral, Kruskal-Wallis test | *H*(2) = 15.37 | 0.0014 |
| 4B (top, left) | Indegree, outside, Kruskal-Wallis test | *H*(2) = 11.82 | 0.0041 |
| 4B (bottom, left) | Outdegree, ipsilateral, Kruskal-Wallis test | *H*(2) = 11.11 | 0.0116 |
| 4B (bottom, left) | Outdegree, outside, Kruskal-Wallis test | *H*(2) = 9.70 | 0.0117 |
| 4B (top, middle) | Authority, ipsilateral, Kruskal-Wallis test | *H*(2) = 15.71 | 0.0006 |
| 4B (top, middle) | Authority, outside, Kruskal-Wallis test | *H*(2) = 17.9 | 0.0006 |
| 4B (top, middle) | Hub, ipsilateral, Kruskal-Wallis test | *H*(2) = 15.09 | 0.0016 |
| 4B (bottom, middle) | Hub, outside, Kruskal-Wallis test | *H*(2) = 11.19 | 0.0056 |
| 4B (bottom, right) | Katz-receive, ipsilateral, Kruskal-Wallis test | *H*(2) = 16.82 | 0.0007 |
| 4B (top, right) | Katz-receive, contralateral, Kruskal-Wallis test | *H*(2) = 9.72 | 0.0116 |
| 4B (top, right) | Katz-receive, outside, Kruskal-Wallis test | *H*(2) = 6.10 | 0.0474 |
| 4B (top, left) | Indegree, ipsilateral, focal vs multifocal, Dunn’s test | *z* = -2.78 | 0.0107 |
| 4B (top, left) | Indegree, ipsilateral, focal vs non, Dunn’s test | *z* = -3.74 | 0.0011 |
| 4B (top, left) | Indegree, outside, focal vs non, Dunn’s test | *z* = -3.43 | 0.0018 |
| 4B (bottom, left) | Outdegree, ipsilateral, focal vs multifocal, Dunn’s test | *z* = -2.32 | 0.0408 |
| 4B (bottom, left) | Outdegree, ipsilateral, focal vs non, Dunn’s test | *z* = -3.20 | 0.0066 |
| 4B (bottom, left) | Outdegree, outside, focal vs non, Dunn’s test | *z* = -3.06 | 0.0066 |
| 4B (top, middle) | Authority, ipsilateral, focal vs multifocal, Dunn’s test | *z* = -3.77 | 0.0005 |
| 4B (top, middle) | Authority, ipsilateral, focal vs non, Dunn’s test | *z* = -2.83 | 0.0094 |
| 4B (top, middle) | Authority, outside, focal vs multifocal, Dunn’s test | *z* = -4.17 | 0.0002 |
| 4B (top, middle) | Authority, outside, focal vs non, Dunn’s test | *z* = -2.16 | 0.0466 |
| 4B (bottom, middle) | Hub, ipsilateral, focal vs multifocal, Dunn’s test | *z* = -3.82 | 0.0008 |
| 4B (bottom, middle) | Hub, ipsilateral, focal vs non, Dunn’s test | *z* = -2.41 | 0.0321 |
| 4B (bottom, middle) | Hub, outside, focal vs multifocal, Dunn’s test | *z* = -3.05 | 0.0068 |
| 4B (bottom, middle) | Hub, outside, multifocal vs non, Dunn’s test | *z* = 2.20 | 0.0414 |
| 4B (top, right) | Katz-receive, ipsilateral, focal vs multifocal, Dunn’s test | *z* = -2.77 | 0.0170 |
| 4B (top, right) | Katz-receive, ipsilateral, focal vs non, Dunn’s test | *z* = -3.96 | 0.0007 |
| 4B (top, right) | Katz-receive, contralateral, multifocal vs non, Dunn’s test | *z* = -2.87 | 0.0170 |
| 4B (top, right) | Katz-receive, outside, multifocal vs non, Dunn’s test | *z* = -2.39 | 0.0383 |
| 5A (bottom, left) | Outdegree, focal, likelihood-ratio test | *LRT*(1) = 11.19 | 0.0025 |
| 5A (top, middle) | Authority, focal, likelihood-ratio test | *LRT*(1) = 11.29 | 0.0023 |
| 5A (bottom, middle) | Hub, focal, likelihood-ratio test | *LRT*(1) = 12.24 | 0.0014 |
| 5A (bottom, right) | Katz-broadcast, focal, likelihood-ratio test | *LRT*(1) = 12.06 | 0.0015 |
| 5A (bottom, left) | Outdegree, focal, within vs outside, *t-*test | *t*(3) = -4.43 | 0.0214 |
| 5A (top, middle) | Authority, focal, within vs outside, *t-*test | *t*(3) = -4.31 | 0.0230 |
| 5A (bottom, middle) | Hub, focal, within vs outside, *t-*test | *t*(3) = -4.66 | 0.0187 |
| 5A (bottom, right) | Katz-broadcast, focal, within vs outside, *t-*test | *t*(3) = -4.59 | 0.0194 |
| 5A (bottom, left) | Outdegree, within, likelihood-ratio test | *LRT*(2) = 11.34 | 0.0069 |
| 5A (bottom, middle) | Hub, within, likelihood-ratio test | *LRT*(2) = 7.57 | 0.0454 |
| 5A (bottom, left) | Outdegree, ipsilateral, focal vs multifocal, Dunn’s test | *t*(15) = -3.30 | 0.0085 |
| 5A (bottom, left) | Outdegree, ipsilateral, focal vs non, Dunn’s test | *t*(15) = -3.23 | 0.0085 |
| 5A (top, middle) | Authority, ipsilateral, focal vs multifocal, Dunn’s test | *t*(15) = -2.52 | 0.0355 |
| 5A (top, middle) | Authority, ipsilateral, focal vs non, Dunn’s test | *t*(15) = -2.52 | 0.0355 |
| 5B (top, left) | Indegree, ipsilateral, likelihood-ratio test | *LRT*(2) = 9.45 | 0.0266 |
| 5B (bottom, middle) | Hub, ipsilateral, likelihood-ratio test | *LRT*(2) = 10.82 | 0.0133 |
| 5B (top, right) | Katz-receive, ipsilateral, likelihood-ratio test | *LRT*(2) = 8.39 | 0.0452 |
| 5B (top, left) | Indegree, ipsilateral, focal vs multifocal, *t-*test | *t*(7) = -2.76 | 0.0421 |
| 5B (top, left) | Indegree, ipsilateral, focal vs non, *t-*test | *t*(7) = -3.15 | 0.0421 |
| 5B (bottom, middle) | Hub, ipsilateral, focal vs multifocal, *t-*test | *t*(7) = -3.66 | 0.0243 |
| 5B (bottom, middle) | Hub, ipsilateral, focal vs non, *t-*test | *t*(7) = -2.66 | 0.0484 |
| 5B (bottom, right) | Katz-receive, focal, likelihood-ratio test | *LRT*(1) = 9.80 | 0.0157 |
| 5B (bottom, right) | Katz-broadcast, focal, likelihood-ratio test | *LRT*(1) = 9.34 | 0.0281 |

FDR: false-discovery rate
